# Visualizing leukocyte trafficking in the living brain with 2-photon intravital microscopy

**DOI:** 10.3389/fncel.2012.00067

**Published:** 2013-01-08

**Authors:** Saparna Pai, Karyn J. Danne, Jim Qin, Lois L. Cavanagh, Adrian Smith, Michael J. Hickey, Wolfgang Weninger

**Affiliations:** ^1^Immune Imaging Program, The Centenary InstituteNewtown, NSW, Australia; ^2^Sydney Medical School, University of SydneySydney, NSW, Australia; ^3^Department of Medicine, Centre for Inflammatory Diseases, Monash UniversityMelbourne, VIC, Australia; ^4^Cytometry and Imaging, The Centenary InstituteNewtown, NSW, Australia; ^5^Discipline of Dermatology, University of SydneySydney, NSW, Australia; ^6^Department of Dermatology, Royal Prince Alfred HospitalCamperdown, NSW, Australia

**Keywords:** inflammation, leukocytes, brain, vasculature, microscopy, trafficking, *in vivo*

## Abstract

Intravital imaging of the superficial brain tissue in mice represents a powerful tool for the dissection of the cellular and molecular cues underlying inflammatory and infectious central nervous system (CNS) diseases. We present here a step-by-step protocol that will enable a non-specialist to set up a two-photon brain-imaging model. The protocol offers a two-part approach that is specifically optimized for imaging leukocytes but can be easily adapted to answer varied CNS-related biological questions. The protocol enables simultaneous visualization of fluorescently labeled immune cells, the pial microvasculature and extracellular structures such as collagen fibers at high spatial and temporal resolution. Intracranial structures are exposed through a cranial window, and physiologic conditions are maintained during extended imaging sessions via continuous superfusion of the brain surface with artificial cerebrospinal fluid (aCSF). Experiments typically require 1–2 h of preparation, which is followed by variable periods of immune cell tracking. Our methodology converges the experience of two laboratories over the past 10 years in diseased animal models such as cerebral ischemia, lupus, cerebral malaria, and toxoplasmosis. We exemplify the utility of this protocol by tracking leukocytes in transgenic mice in the pial vessels under steady-state conditions.

## Introduction

### Background

The immune system is a highly complex and dynamic network of immune cells that travel throughout the body under both normal and inflammatory conditions (Matzinger, 1994; Kunkel and Butcher, 2003; Germain et al., 2006; Cahalan and Parker, 2008; Littman and Rudensky, 2010; Randolph, 2011; Ousman and Kubes, 2012; Victora and Nussenzweig, 2012). It is therefore important to understand how immune cells are recruited to organs and how they behave after their extravasation into specific tissue microenvironments, including the brain (Cahalan et al., 2002; Ransohoff et al., 2003; Mrass and Weninger, 2006; Hickey and Kubes, 2009; Mrass et al., 2010; Wilson et al., 2010; Ousman and Kubes, 2012; Ransohoff and Engelhardt, 2012; Sallusto et al., 2012). While epifluorescence microscopy based approaches have been used for a long time to study leukocyte-endothelial interactions (von Andrian, 1996; Engelhardt and Wolburg, 2004; Sumen et al., 2004; Engelhardt and Ransohoff, 2005; Teixeira et al., 2010), newer techniques of optical imaging such as 2-photon intravital microscopy (2P-IVM) has recently expanded the scope for studying activities of immune cells such as leukocytes within the central nervous system (CNS) (Helmchen and Denk, 2005; Mrass and Weninger, 2006; Bartholomaus et al., 2009; Padmanabhan et al., 2010; Wilson et al., 2010; Harris et al., 2012). Analysis of not only how cells are recruited to the CNS but also how they navigate through the brain parenchyma and interact with other cells, stromal and neuronal structures as well as pathogens has provided a whole new vista of information for immunologists, neuroscientists, and clinicians alike (Mrass and Weninger, 2006; Bartholomaus et al., 2009; Coombes and Robey, 2010; Kawakami and Flugel, 2010; Wilson et al., 2010; Amornphimoltham et al., 2011; McGavern and Kang, 2011).

Due to easier accessibility to 2-photon imaging systems in recent times, intravital imaging of the CNS is fast becoming the preferred modality for *in vivo* analysis of brain and spine tissue (Siffrin et al., 2010; Herz et al., 2011) fuelling a demand for simple and reliable descriptive imaging protocols as well as affordable equipment for mouse preparation. In this paper, we provide a 2P-IVM-based intravital brain-imaging (2P-IBI) model that addresses some of these requirements. The protocol primarily aims to provide a practical guide for investigators new to the brain-imaging field. It is optimized for tracking the behavior of leukocytes within the vasculature of the brain but can be easily adapted however to address varied CNS-related biological questions in the living brain.

### Description of the model

Our 2P-IBI model uses a cranial window preparation that is best suited for short imaging sessions of up to 1.5 h. Preparatory time for a routine session will take 70 min, with data acquisition of approximately 6 mm of surface area of the cerebral cortex taking ~1.5 h. Where imaging must be extended beyond 1.5 h, 2P-IBI can be tailored for longer sessions of up to 6 h. For this we provide add-on steps in the protocol, involving the installation of a superfusion chamber that continuously pumps artificial cerebrospinal fluid (aCSF) over the exposed brain tissue and simulates the normal brain microenvironment (James et al., 2003; Lister and Hickey, 2006; Norman et al., 2008; Wong et al., 2008; Nie et al., 2009). An hour needs to be allocated for the functional operation of the superfusion chamber thus taking the total preparatory time to approximately 2 h. The design and dimensions of the equipment used in 2P-IBI are suited for mice but can be adapted for other rodents such as rats. The protocol provides a comprehensive list of equipment, reagents, and procedures required for setting up a similar model so that a non-specialist can acquire this capability simply by implementing this report. It also guides in the identification and management of potential trouble spots that arise throughout the procedure.

### Advantages of the model

The stereotaxic frame used in 2P-IBI for conducting surgical procedures in mice has custom-built designs that render it user-friendly and cost-effective. This excludes having to purchase and modify potentially bulky commercial stereotaxic frames offered by most manufacturers. We provide the design and dimensions for building such a frame. The frame is designed to fit within the small area typically available between the nose of the dipping objective and the stage of the microscope. Although the technique is optimized for leukocyte imaging, 2P-IBI can find varied applicability including for the study of the microenvironment of solid tumors (Yuan et al., 1994), microglial function (Davalos et al., 2005), and amyloid plaque deposition in Alzheimer disease (Robbins et al., 2006). 2P-IBI does not require extensive surgical preparation for absolute sterility as it is performed on anesthetized non-recovery animals. The short preparation time of ~70 min is a particularly useful feature for studying diseased animal models where manifestation of clinical symptoms within a very narrow time “window” can impose rigid time limits for data acquisition. The cranial window preparation used in 2P-IBI however requires removal of the skull, rendering the brain susceptible to pressure and temperature changes over time, hastening the decline of the animal and reducing recording time (Yoder, 2002). More importantly, even a small drop in body temperature is sufficient to reduce leukocyte motility and behavior resulting in confounding artifacts in acquired data (Li et al., 2012). Therefore where imaging is expected to extend longer than 1.5 h, we recommend installation of our superfusion chamber wherein the dura is excised while the brain is maintained under a continuous intracranial pressure (ICP) of 5–8 mm Hg. Thus 2P-IBI serves the requirements of both short and long recording sessions.

The quality of the cranial window preparation in 2P-IBI enables the resolution simultaneously of several different fluorescently labeled components within the CNS, including the pial and cerebral microvasculature, fluorescently labeled red blood cells, platelets and leukocytes as well as non-labeled structures such as collagen fibers using second harmonic generation (SHG) signals. Penetrance of ~250–300 μm into the cerebral cortex allows these structures to be visualized within the deeper regions of the brain. The protocol is particularly useful for (1) characterization of leukocyte migration, behavior and cross-talk and the molecular mechanisms that underlie such processes (Bartholomaus et al., 2009) (2) dissection of immune-surveillance mechanisms in healthy brain tissue (Nimmerjahn et al., 2005) (3) in-depth analysis of pathogen-leukocyte interaction and identifying the precise time point when immune responses are initiated *in vivo* (Hickman et al., 2009; Coombes and Robey, 2010) (4) study of immune-evasion mechanisms of pathogens (Kamerkar and Davis, 2012) and last but not the least (5) understanding the impact of the stromal and neural components of the brain microenvironment on the host immune response (Constantin et al., 2009; Wake et al., 2009).

### Limitations of the model

During inflammation, leukocytes roll along the endothelial wall of the blood vessel up to 20–50 μm/sec (Carvalho-Tavares et al., 2000; Pai et al., unpublished observations). The limited scan speed of a point line scanner as used in our microscope setup can be restrictive while tracking these rolling cells. Tracking of rolling cells can be achieved however by scanning a single Z plane. While this strategy is useful, loss of information from adjoining Z planes can undermine an important advantage 2P-IBI offers—generation of high-resolution four dimensional (x,y,z,t) images. Further, collecting brain images from a single Z plane can augment the effect of “bobbing,” a ripple effect of respiration-induced movements on the brain (Belluscio, 2005). The movement can create drift in the image where it is difficult to reproducibly scan the same Z plane at each time interval. To circumvent some of these limitations, a trigger box system that times the image acquisition to the pulse of the animal can be used. Another way around this problem is to use high-speed acquisition modalities offered by some 2-Photon manufacturers such as LaVision BioTec's Trimscope series. This system provides greater scan speed by parallelization of the excitation process through the use of reflective mirrors to split the laser beam into a line of up to 64 beamlets (http://dx.crossref.org/10.1529%2Fbiophysj.106.102459).

### Comparison with other methods

There are primarily two types of cranial window preparations used for live brain imaging: (1) A thinned skull window preparation generally used for imaging large structures such as the cortex, amyloid plaques, and blood vessels that can also be used for imaging leukocytes (Fabene et al., 2008; Yang et al., 2010; Grutzendler et al., 2011) and (2) A closed cranial window preparation suited for imaging neuronal structures as well as for imaging leukocytes (Mostany and Portera-Cailliau, 2008; Cabrales and Carvalho, 2010). 2P-IBI uses a closed cranial window preparation that circumvents the main limitation of using thinned skull preparations-spherical aberration, reduction in 2-photon excitation or distortion in fluorescence emissions due to non-uniformity in the thinned skull (Yang et al., 2010). However a closed cranial window preparation has its own disadvantages—(1) removal of the skull rendering the brain susceptible to pressure and temperature changes over time leading to confounding artifacts (Yoder, 2002) and (2) induction of significant inflammation requiring the use of anti-inflammatories/antibiotics or a waiting period post-surgery before experiments can be initiated (Pan and Gan, 2008; Holtmaat et al., 2009; Yang et al., 2010). 2P-IBI overcomes these disadvantages by using a two-part approach—a closed cranial window preparation is used while imaging for short periods of <1.5 h and a superfusion chamber is installed as an add-on step while imaging for extended periods for up to 6 h. The chamber simulates the brain microenvironment and prevents activation of leukocytes despite the exclusion of anti-inflammatory agents. This model has been collated together based on our experience in studying immune responses *in vivo* within the skull bone marrow (Cavanagh et al., 2005) and CNS (Mrass and Weninger, 2006; John et al., 2009, 2010, 2011; Wilson et al., 2009, 2010; Harris et al., 2012) as well as in the study of disease models such as cerebral ischemia (Wong et al., 2008), lupus (James et al., 2003; Lister and Hickey, 2006; Norman et al., 2008), toxoplasmosis (John et al., 2009; Wilson et al., 2009, 2010), and leishmaniasis (Ng et al., 2008) among others.

## Experimental setup—points to consider

### Fluorophores

Generally, existing guidelines for confocal microscopy suffice when selecting fluorophores for 2P-IBI (for guidelines refer to Hibbs, 2004). It is important to evaluate whether the excitation wavelength of the fluorophore is within the tunable range of the laser. Enhanced green fluorescent protein (GFP) is one of the most effective fluorophores followed by its yellow (YFP) and cyan (CFP) variants. Where multiple cell types need to be detected choose a combination of fluorophores that can be simultaneously excited with minimal spectral emission overlap. The mouse dural membrane, unlike rats, is transparent and permeable to signals emitted from most fluorophores including GFP (Sigler and Murphy, 2010). For fluorophores to which the dura is impermeable such as voltage sensitive fluorescent dyes (VSDI) (Grinvald and Hildesheim, 2004), we recommend installation of our superfusion chamber wherein the dura can be excised. Visualizing endogenously fluorescent cells in transgenic reporter mice is the most attractive approach for visualizing the trafficking of cells *in vivo* (Mempel et al., 2006). Alternately, cells can be purified, labeled with permeable dyes such as CMTMR [5-(and-6)-(((4-Chloromethyl)Benzoyl)Amino)Tetramethylrhodamine], rhodamine 6G, CFSE (Carboxyfluorescein diacetate succinimidyl ester) or with antibody conjugated to dyes such as the Alexa-Fluor series and transferred into recipient mice for visualization (Ng et al., 2011).

### Leukocyte-mediated inflammation

Immune cells such as dendritic cells (DC), neutrophils, macrophages, and monocytes form the first line of defense against pathogens (Auffray et al., 2007). These innate cell types are the first to be recruited and/or activated (in a timespan of minutes) at the site of inflammation, infection or injury (Ng et al., 2008, 2011). Any study of leukocytes that involves an invasive surgical procedure therefore must consider the risk of non-specifically recruiting the very immune cells that are the subject of investigation. Trauma-induced recruitment of immune cells to the surgical site can potentially compromise data interpretation and jeopardize the study. Similarly, a small drop in body temperature is sufficient to reduce leukocyte motility and behavior (Li et al., 2012) resulting in confounding artifacts in acquired data. In our experience, approximately 5% of the animals have to be excluded from further experimentation/analysis due to inflammation induced from the surgical procedure. The normal brain and CNS vasculature does not support rolling and firm adherence of leukocytes (Carvalho-Tavares et al., 2000). As such, induction of rolling and adhesion in the pial microvasculature serves as an internal control in our studies for the induction of inflammation due to animal preparation. As an additional quality control, we have analyzed leukocyte recruitment post-surgery using confocal microscopy. Under optimal conditions, we found no evidence of leukocyte recruitment in either the operated or non-operated hemispheres of formalin-fixed whole mount sections of the brain (data not shown).

### Brain tissue homeostasis

The brain tissue is highly sensitive to subtle changes in its microenvironment. Studies have reported that the mere opening of the dura and exposure of the brain to atmospheric pressure can result in swelling and protrusion of the brain through the window (Kawamura et al., 1990). Further, the CSF has a “sink action” by which the products of brain metabolism such as CO_2_, lactate and hydrogen ions are removed as CSF gets absorbed into the blood stream (Ropper et al., 2005). Even the slightest retention of CO_2_ therefore raises the blood P_CO2_ and correspondingly causes a decrease in the pH of CSF resulting in potent vasodilation, increased cerebral blood flow and intracranial pressure (ICP) (Ropper et al., 2005). Conversely an increase in the pH of CSF can cause a decrease in ICP. In the technique described herein, the integrity of the dura is retained for short imaging sessions of up to 1.5 h. Retaining the dura helps minimize alteration in vascular dynamics or brain tissue homeostasis (Kawamura et al., 1990; Holtmaat et al., 2009). In line with this, the diameter of 28 pial arterioles and venules (0.14 mm^2^ total area) studied remained virtually unchanged throughout the observation period post-surgery (Pai et al., unpublished observations). We do not have enough data to provide information on the stability of this preparation for imaging sessions longer than 1.5 h duration. Where imaging sessions are expected to last longer than 1.5 h, we install the superfusion chamber. In this approach, the dura is excised and the aCSF circulating through the chamber is used to maintain the brain under a continuous ICP of 5–8 mm Hg. The chamber also maintains optimum pH and gas tension by constant bubbling of 12% O_2_, 5% CO_2_, and 83% N_2_ through aCSF where imaging sessions will extend up to 6 h (James et al., 2003; Lister and Hickey, 2006; Norman et al., 2008; Wong et al., 2008; Nie et al., 2009).

### Breach in BBB

The blood–brain barrier (BBB) is a neurological unit that maintains the “immune privilege” status of the brain by restricting the passage of solutes as well as the extravasation of cells and pathogens from the blood vessel into the parenchyma (Streilein, 1993; Mrass and Weninger, 2006; Engelhardt and Coisne, 2011; Engelhardt and Ransohoff, 2012; Masocha and Kristensson, 2012; Takeshita and Ransohoff, 2012). In line with this, leakage of dextran rhodamine was not observed from blood vessels of healthy C57BL/6 mice that underwent our 2P-IBI procedure (Saria and Lundberg, 1983 and data not shown; Kawamura et al., 1990). However in diseased animal models of the CNS such as experimental autoimmune encephalitis (EAE) or experimental cerebral malaria (ECM) (Renia et al., 2012), a breach in the BBB was associated with leakage of vascular probes into the parenchyma. Where the clinical disease is associated with a breach in BBB, low molecular weight vascular probes such as Evans blue (which binds to albumin following intravenous administration) or Na^+^–fluorescein are suboptimal as their low molecular weight facilitates their extravasation (Saria and Lundberg, 1983; Kawamura et al., 1990; Renia et al., 2012). We use high molecular weight dextran in the inflamed brain (MW 2,000,000), as they are lower sensitivity indicators of barrier damage. Vascular probes must always be administered intravenously (i.v) after all surgical procedures and associated bleeding has come to a complete stop as persistent bleeding will allow vascular probes to leak out and obscure the cortical structures.

### Movement

There are several sources of movement that can impact on the quality of the brain image during data acquisition. A regular small amplitude pulsatile movement ranging from 2–5 Hz (cycles/second) that is synchronized to the heartbeat can reflect in the image (Belluscio, 2005; Holtmaat et al., 2009; Yang et al., 2010). Large amplitude respiration-induced movements caused by chest motion during breathing can also cause drifts in the image. Our studies have determined that there are primarily two parameters that influence these movements: (1) state of anesthesia of the experimental mice and (2) effective head restraint. (Note: Descriptive procedures and a troubleshooting guide are provided herein on how to avoid these movement artifacts).

## Materials

### Reagents

- Artificial cerebrospinal fluid (132 mM NaCl, 2.95 mM KCl, 1.71 mM CaCl_2_.2H_2_0, 1.4 mM MgSO_4_, 6.7 mM Urea, 24.6 mM NaHCO_3_, 3.71 mM Glucose, pH 7.4) (aCSF must be pre-warmed to 37°C)
- Dental glue (Vertex, Cat No. XY244L01)
- Bone wax (Lukens, Cat No. 2009-05)
- Accelerant (Loctite 406, Cat No. 40633)
- Dextran rhodamine (Invitrogen, Cat No. D7139)
- Normal saline (Baxter Healthcare, Cat No. F8B123)
- Hydrogen peroxide (Merck, Cat No. 10366)
- High vacuum silicone grease (Sigma, Cat No. MKBD 9670)
- Gelfoam (Pfizer, Cat No. 09-0891-04-015)
- PBS (potassium phosphate monobasic 0.2 g per liter, potassium chloride 0.2 g per liter, sodium chloride 8.0 g per liter, sodium phosphate dibasic (anhydrous) 1.15 g per liter)
- Ketamine (Cenvet, Cat. No. K1000)
- Xylazine (Cenvet, Cat. No. X5010)
- Buprenorphine (Cenvet, Cat. No. T9840)

### Mice

Procure 6–8-week old mice. Mice strains tested for this protocol includes ROSA26 (Muzumdar et al., 2007), DPE-GFP (Mempel et al., 2006), CD11c-YFP (Lindquist et al., 2004), and Macgreen (Sasmono et al., 2003) all on a C57BL/6 background. All procedures described here were approved either by the University of Sydney Animal Ethics Committee or the Monash Medical Centre Animal Ethics Committee.

**WARNING!** All experimental work involving live animals requires official approval from the institutional and/or regional animal ethics committee.

### Reagent set-up

#### Ketamine, xylazine, and buprenorphine

Administer Ketamine at 100 mg/kg of body weight and Xylazine at 10 mg/kg of body weight. A freshly prepared mixture of Ketamine and Xylazine provides effective anesthesia and analgesia for 20–30 min. Administering a single dose of Buprenorphine, a non-steroidal opiate at 100 μg/kg of body weight provides lasting pain relief with minimal side effects. To check its anesthetic state, the experimental mouse must be monitored regularly every 5 min for awareness signs such as whisker twitching, palpebral (blink) reflex, pedal withdrawal reflex and respiration rate. Surgical procedures must begin only after the animal enters a deep state of anesthesia. Booster doses of Ketamine at 30 mg/kg of bodyweight and Xylazine at 3 mg/kg of body weight can be administered as required.

**WARNING!** Ketamine and Buprenorphine are narcotic drugs that must be handled according to institutional and local safety regulations.

### Equipment

- Cover glass (HD Scientific, Cat No. HD LD2222 1.01P0)
- Cotton balls (Johnson & Johnson)
- Curved splinter forceps (WPI-World Precision Instruments, Cat No. 14187)
- Scissors (WPI, Cat No. 14393)
- Scalpel (WPI, Cat No. 500236)
- Scalpel blade (Allgaier Instrumente, Cat No. 02-040-015)
- Animal heat pad (Fine Science Tools, Cat No. 21060-00)
- Rectal probe (Fine Science Tools, Cat No. 21060-01)
- Ear Bars (WPI, Cat No. 502056)
- Peristaltic pump (John Morris Scientific, Cat No. F155001, F117604)
- Thermostat TR-200 (Fine Science Tools, Cat No. 21052-00)
- Polyethylene tubing (BD, Cat No. 427420)
- Cotton tipped applicators (Puritan Medical Products company, Cat No. 427420)
- Omni drill 35 (WPI, Cat No. 503599)
- Burs & Bits (WPI, Cat No. 503599)
- Phillips head screwdriver (JBS, Cat No. 6768098)
- Tape (Hystick)
- Blotting paper (Whatman, Cat No. 3030917) (cut into 2″ × 2″ pieces)
- Insulin syringe needle (BD, Cat No. 326105)
- Transfer pipettes (Biologix Research Company, Cat. No. 30-0135)
- Dissection microscope (Leica M80, Leica)
- Petridish (BD, Cat. No. 353002)
- Compressed gas, Oxidising NOS (BOC)
- Kimwipes (Kimtech Science, cat. no. 34155)
- Heavy crepe bandage (ACCO Australia, 105109) (6″ pieces serve as cotton blanket)
- 30 G needle (BD, Cat. No. 304000)
- 18 G needle (BD, Cat. No. 305211)
- 5 ml syringe (Terumo, Cat. No. SS05S)
- Heating block (RATEK instruments, Cat. No. DBH10)

### Microscope

- Optional: TriMScope II single-beam 2-photon microscope (LaVision BioTec)
- Optional: Tunable (680–1080 nm) MaiTai HP lasers (Spectra Physics) (≥3.3 W at 800 nm; pulse length of 140 fs, 80 MHz repetition rate)
- Water-dipping objectives (20×, NA = 0.95; XLUMPLFLN 20 × W) (Olympus)
  **IMPORTANT!** Clean with lens cleaning wipes after every use.
- Photomultiplier module blue (quantum efficiency = 17% at 400 nm; Hamamatsu, Cat. no. H 6780-01)
- Photomultiplier module green/red (QE = 15% at 630 nm; Hamamatsu, Cat. no. H 6780-20)
- High-sensitivity photomultiplier module (QE = 40% at 550 nm; Hamamatsu, Cat. no. H 7422-40)
- AHS LAMP 12 V/100 W halogen lamp for wide-field epifluorescence
- Optical table (Newport Corporation)

### Microscope filter and mirror sets

**Set A:** 495 long-pass (LP; Chroma, cat. no. T495LPXR), 560 LP (Chroma, Cat. no. T560LPXR), 475/42 band-pass (BP; Semrock, cat. no. FF01-475/42-25), 525/50 BP (Chroma, Cat. no. ET525/50m), and 665/40 BP (Chroma, Cat. no. NC028647). This filter set is used for imaging GFP, SHG, and TRITC. A Maitai laser of 900 nm wavelength is used for exciting GFP and TRITC.
**Set B:** 520 LP (Semrock, Cat. no. FF520-Di02-25×36), 650 LP (Chroma, Cat. no. 640 DCLP), 475/42 BP, 593/40 BP (Semrock, Cat. no. FF01-579/ 34-25), and 665/40 BP. This filter set is used for imaging tdTomato and SHG. A Chameleon laser of 800 nm wavelength is used for SHG and OPO 1080 nm is used for exciting tdTomato.
**Set C:** 495 LP, 520 LP, 560 LP, 475/42 BP, 525/50 BP, and 665/40 BP. This filter set is used for imaging YFP, SHG, and TRITC. A Maitai laser of 960 nm wavelength is used for exciting YFP and a Chameleon laser of 880 nm is used for exciting TRITC.

### Equipment set-up

#### Stereotaxic frame

**Figure 1:**
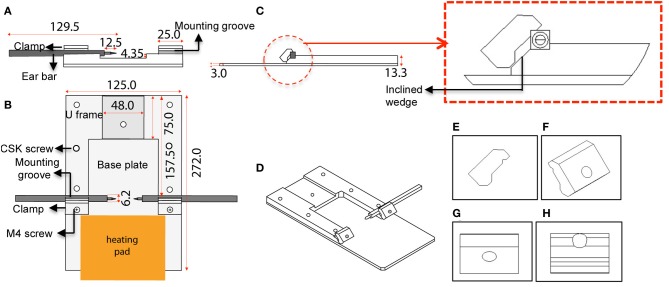
**Schematic diagram and dimensions of the custom-designed stereotaxic frame used in the 2P-IBI model. (A)** Section view. Cross section view shows the mounting groove engaged with an ear bar. **(B)** Top view. Position of the heavy base plate, U frame, 2 clamps, 6 CSK screws as well as the 2 M4 screws are shown. Position of the heating pad is depicted. **(C)** Side view. Thickness of the base plate and U frame are shown. Red circle shows a clamp gripping an ear bar. Red box shows a magnified view of the clamp and the inclined wedge gripping the ear bar. Note the notch of the ear bar slips into a groove in the underbelly of the clamp. **(D)** 3-dimensional view of the frame. **(E–H)** Angle views of the clamp. **(E)** Side view with grooves in the underbelly, **(F)** 3-dimensional view with a hole for fitting the M4 screw, **(G)** Top view, **(H)** Ridges carved on the inclined wedge to grip the ear bar.

**Figure 2:**
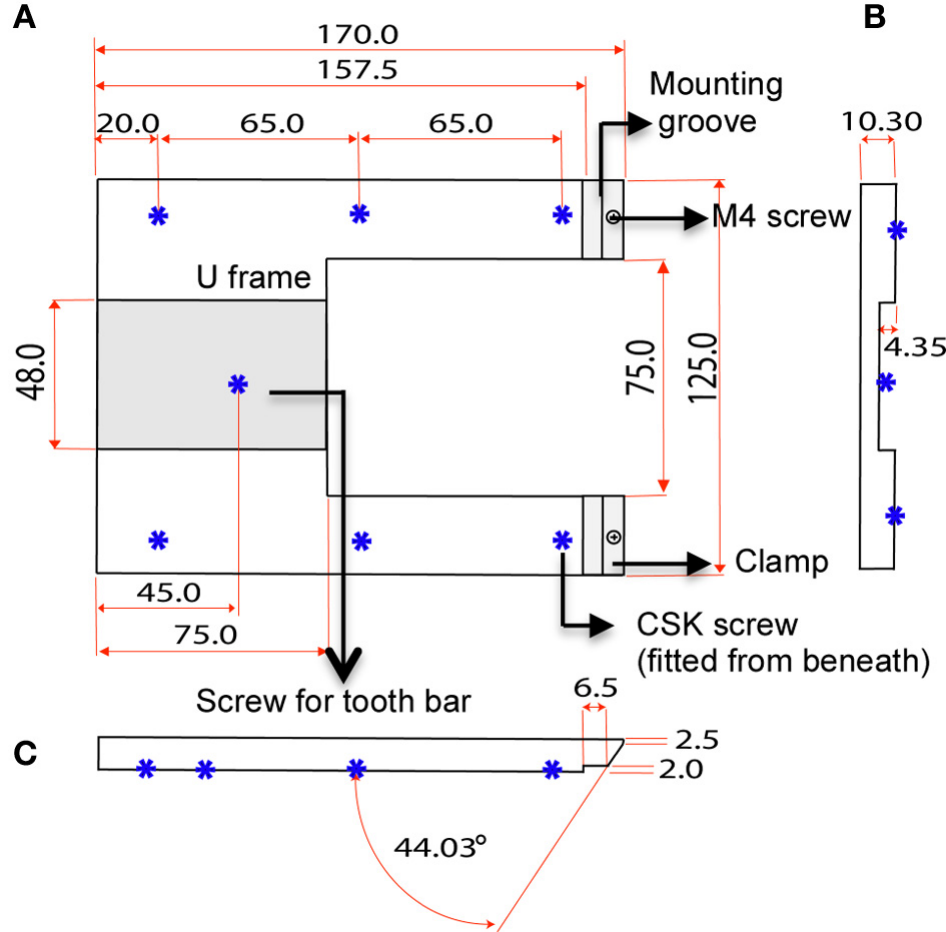
**Dimensions of U frame. (A)** Top view shows the position of the clamps and 6 CSK screws fitted from beneath. The screws bolt the “U” frame to the base plate. **(B)** Section view shows the concavity of the U frame that makes provision for a tooth bar. **(C)** Side view shows the angle of the clamp in relation the U frame.

The custom-designed stereotaxic frame used in this protocol consists of a heavy aluminium base plate, “U” frame and 2 ear bar clamps and is most suited for 6–8-week old mice (Figures 1, 2). The bulky base helps to provide stability for operator use. The “U” frame is bolted to the base plate via 6 CSK screws fitted from beneath. The clamps are then fitted in place using M4 CSK screws. Ear bars with 18° or 45° points may be used. The 18° ear bars provide good head restraint but can cause internal ear hematoma and acute inflammation (Prestwich et al., 2008). We use the non-rupture wide angle 45° points that provide adequate head restraint and carry minimal risk of injury or inflammation. Mount ear bars into the mounting groove. Once assembled, the contraption can be stored in this configuration. A tooth bar and nose clamp did not provide additional stability for head restraint in our experiments.

**IMPORTANT!** Measure the distance between the nose of the dipping objective and the stage of the microscope. The stereotaxic frame must fit within the measured distance.

**IMPORTANT!** The frame and ear bars form a crucial contraption for restraining the head and will ultimately determine the quality of the brain images. This step requires surgical practice.

#### Electronically regulated heating pad

To maintain core body temperature, place the mouse on a heating pad that is maintained at a constant of 37°C. Use a feedback rectal probe to record the body temperature and regulate the heating pad at 37°C. Set the thermostat at 37°C.

#### Circulating superfusion chamber

**Figure 3:**
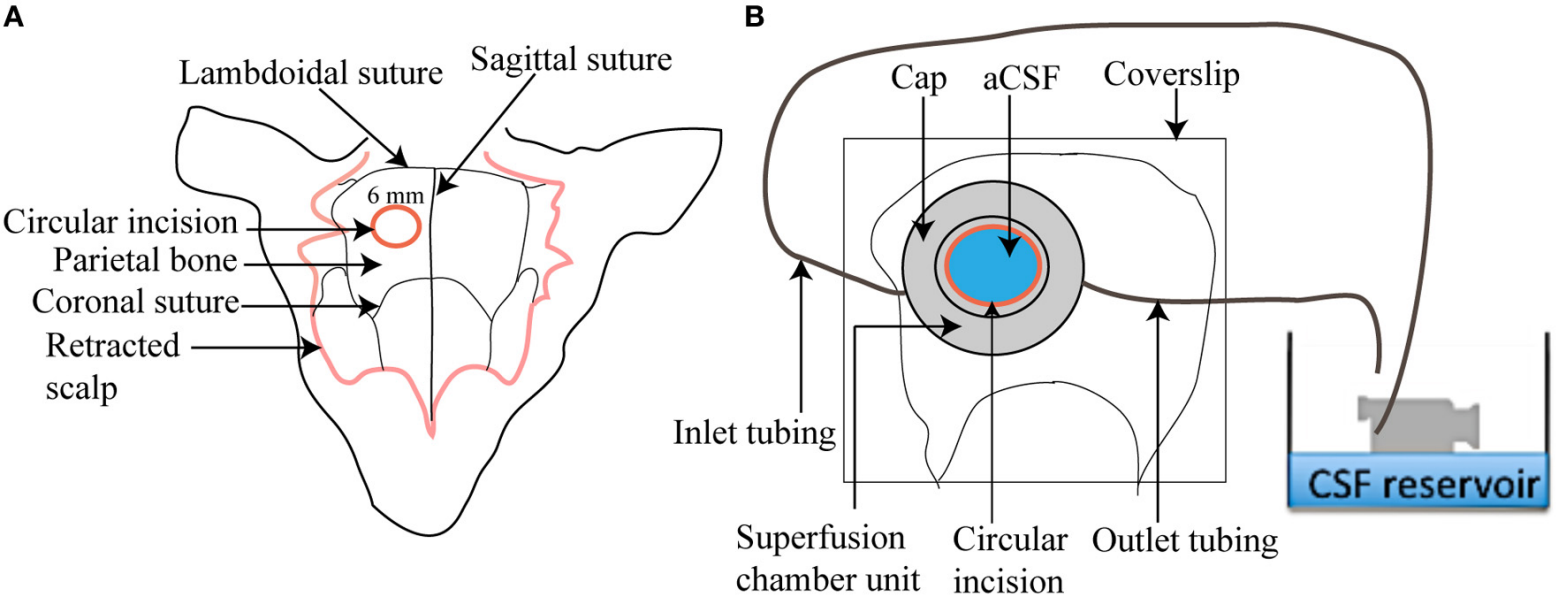
**Illustration of a superfusion chamber. (A)** A circular incision of 6 mm is made in the parietal bone after scalp retraction to form a cranial window **(B)** A magnified view of a superfusion chamber that has been glued to the cranial window. An inlet and outlet tubing is connected to the chamber to circulate warm aCSF over the cranial window.

The circulating superfusion chamber unit consists of a cap-like reservoir, PVC tubing, pump-operated circulating water bath, N_2_/CO_2_/O_2_ cylinder, and a beaker containing aCSF (Figure 3). The custom designed stainless steel cap-like reservoir is 2 mm high with an internal diameter of 7 mm. It has an outer rim 0.4 mm wide and 15 mm in diameter extending horizontally from the chamber. It is modified to include a concavity 8 mm in radius that will mold to the shape of the mouse skull. The chamber contains two ports, one for attachment of inlet polyethylene tubing for superfusing the brain surface with aCSF and the other for attachment of outlet polyethylene tubing that will collect aCSF after superfusion. The outlet tubing is positioned at an elevation of 10 cm above the mouse brain to maintain intracranial pressure at 5–8 mm Hg throughout the session. To begin, freshly prepared aCSF is decanted into a small beaker and placed in a peristaltic pump-operated warm circulating water bath and pre-warmed to 37°C. The operational speed of the peristaltic pump is adjusted to maintain aCSF infusion at 0.3 ml/min. The aCSF is continuously bubbled with a mixture of 12% O_2_, 5% CO_2_, and 83% N_2_.

**IMPORTANT!** Assemble the unit and keep it ready to save on operational time.

## Procedure

For troubleshooting guide see Table 1 below.

**Table 1:** **A troubleshooting guide to manage potential trouble areas**.

**Expected trouble spots**			
**Number**	**Problem**	**Possible explanation**	**Solution**
3	Body temperature reflected by the thermostat is unstable or is not at 37°C	Rectal thermistor is improperly inserted or has dislodged	Check that the rectal thermistor is properly inserted. If dislodged, re-secure the probe to the heat pad with tape. Check that the thermostat is set at 37°C
3	Animal not anesthetized after 15 min	Variability due to age, size, or disease status	Administer a booster dose
3	Animal has increased heart rate and/or a weak pedal withdrawal reflex but is otherwise asleep	Anesthetic has lost some of its potency	Anesthetics must be freshly prepared and stored in lightproof containers
		Variability due to age, size, or disease status	Administer a booster dose
3	Animal begins to awaken as evident from twitching of whiskers	20–30 min have lapsed since administration of anesthetic	Administer a booster dose
7	Ear bars cannot engage the ear canal	Head is resting at an angle	Rest the head in a horizontal position on the paper stack
		Head is higher or lower than the horizontal axis of the ear bars	Add or remove pieces of blotting paper a few mm at a time to get the axis of the horizontally mounted ear bars to the same level as the ear canal
			Use 6–8-week old mice, as the frame is most suited for restraining smaller sized animals
7	Increased heart rate during ear bar engagement	Animal inadequately anesthetized	Provide more time for anesthetic action and if that fails, administer a booster dose
7	Animal stops breathing during or after ear bar engagement	Diaphragmatic paralysis caused by impingement of the vagal nerve	Release ear bars immediately. Ensure head is horizontally positioned and jaw supported by the paper stack. Once breathing is regular and stable, continue
8	Animal hair is contaminating the surgical area	Hair is falling loose	Hair must be liberally wetted before making an incision. Scalp must be taped to the pad or can be removed altogether from over the surgical region
9	Connective tissue entangles with the burr during the drilling process	Periosteum and adjoining connective tissue have not been completely removed	Retract or remove any part of the scalp that can potentially come in contact with the drill
			Use a scalpel and cotton applicators dipped in H_2_O_2_ to scrape and remove any remnants of connective tissue
10	Skull or dura is pierced while drilling	Drill positioning is incorrect	Hold drill at an angle of 25° to avoid piercing the skull or dura
		Excessive pressure applied while drilling	Avoid putting pressure on the bone while drilling
			Repeat surgical procedure on a new mouse as the dura is likely to be damaged
11	Bleeding	Damage to blood vessel by forceps	Forceps must be introduced horizontally at an angle of 10°. Do not push or insert into the window
		Damage to blood vessel while lifting the cranial flap	Flap must not be pulled or tilted at an angle while lifting as its sharp edge can damage blood vessels
		Damage to blood vessel by the drill	Hold drill at an angle of 25° to avoid piercing the skull or dura. For all of the above, apply gelfoam to soak up the blood; minor bleeding must stop in 3–4 min. If severe bleeding occurs, continuing with the procedure must be re-evaluated
11	Brain bulges through the craniotomy	Skull or dura is pierced while drilling exposing the brain to atmospheric pressure	Repeat surgical procedure on a new mouse as the dura is likely to be damaged
11	Dura is lifted with the flap	Excessive pressure applied while drilling resulting in a deep incision	While chipping, just touch drill to the bone. Chip the bone gently over and over again at low speed until the bone just gives way
			Repeat surgical procedure on a new mouse as the brain is likely to bulge
12	Cap does not sit firmly on the skull or is easily dislodged	Bone wax has cracked while rolling or encircling the cap	Pre-warm the wax and try rolling again into a thin continuous sausage
12	Bone wax comes into contact with the circular incision	This can occur without impacting on the procedure	Use forceps to clear bone wax from the circular incision so that it just covers the outside edge
13–14	Cyanoacrylate glue/accelerant comes into contact with the circular incision inside the cap	Stick picks up excessive glue/accelerant	Make sure stick picks up just enough so glue/accelerant drops off the pointy end slowly one drop at a time
		Bone wax is not properly molded around the cap so glue/accelerant leaks into the inside of the cap	The sausage must encircle the base of the cap completely and must be molded with forceps so that it seals the joint between the cap and the skull. If the problem persists then repeat surgical procedure on a new mouse
13–14	Cyanoacrylate glue/accelerant floods the outside of the cap	Excessive glue/accelerant applied to the outside of the cap	Make sure just enough glue/accelerant is applied on the outside of the cap and the stick is pointy to facilitate easy application Mop up any excess glue with kimwipes
		There is an interval between application of glue and accelerant	Apply the accelerant immediately after applying the glue
			Mop up any excess accelerant immediately with kimwipes
15	Superfusion chamber leaks	Bone wax is not properly molded around the cap	The sausage must encircle the base of the cap completely and must be molded with forceps so that it seals the joint between the cap and the skull
		Glue action is not adequate	Glue must completely encircle the outside of the cap so that it fortifies the joint between the circular sausage and the skull
		Accelerant action is not adequate	In order to act on the glue, accelerant must completely encircle the glue on the outside of the cap
			If not dry, accelerant can be left for a further 2–3 min
15–16	aCSF circulates slowly or not at all through the chamber	One of the two ports is blocked by bone wax	The two ports must be clear of wax at all times
		Inlet or outlet tubing is displaced	Make sure tubing is in place
		Inlet or outlet tubing has bubbles	If bubbles are obstructing flow then allow aCSF to run out until bubbles are voided. Reattach tubing
		Speed of the peristaltic pump is not optimal	Check the operational speed of the peristaltic pump is set at 0.3 ml/min
17	Bleeding	Damage to blood vessel while retracting the dura	Use a dissection microscope to select a vascular region so that the dura is visible. Make a small incision and the dura will retract. Minor bleeding must stop in 3–4 min. If excessive bleeding occurs, continuing with the procedure must be re-evaluated
24	Small amplitude pulsatile movement	Heartbeat impacting on the image	Keeping the exposed brain region to ≤6 mm and using a large, heavy coverglass can minimize impact
24	Large amplitude respiration-induced movement	Animal not effectively anesthetized	Administer a booster dose
		Animal begins to awaken from anesthesia	Administer a booster dose
		Head not effectively restrained by ear bars	Immobilize the head again. This is not always possible in which case surgical procedure must be repeated on a new mouse
25	Blood flow is sluggish	Core body temperature of the mouse is dropping	Check that the rectal thermistor is properly inserted. If dislodged, re-secure the probe to the heat pad with tape. Check that the thermostat has been set at 37°C. Cover the mouse with cotton blanket
25	Leukocytes recruited into the blood vessels migrate slowly while interacting with the endothelium	Inflammation has been induced during the surgical procedure	Repeat surgical procedure on a new mouse
25	Fluorescence signal is weak	Voltage of PMT is low	Increase the PMT voltage again
		Laser power is too low	Increase the laser power
		Inappropriate choice of excitation wavelength	Try other excitation wavelengths or a different dye
		Inappropriate choice of emission filter or dichroics	Review filter selection and try alternative
		Laser misaligned	Have microscope system checkout and/or serviced
25	Fluorescence signal of vascular probe is weak	Integrity of the BBB is compromised during the preparation and vascular probe has leaked out	Continuing with the data acquisition must be re-evaluated
		Intravenous injection not successful	Repeat injection
		Inappropriate choice of excitation wavelength	Try other excitation wavelengths or a different dye

### Presurgical preparation • 25 min

1. Transfer cells/leukocytes i.v through the tail vein. Administer bacteria, parasites, or other agents if any by the preferred route.
  **IMPORTANT!** i.v injections may require pre-warming of mice to dilate the tail vein. It is difficult to pre-warm mice once the surgical procedure is initiated. Administration of cells via the i.v route must therefore be completed before proceeding to the next stage.
  **IMPORTANT!** Mice pre-warmed for procedures such as i.v injections are prone to surgery-induced bleeding. Unless hypothermic pre-warmed mice must be allowed to recover at room temperature prior to surgery.
2. Stick the electronically regulated animal-heating pad on to the stereotaxic frame securely with tape. Connect the leads and turn on the thermostat. The pad will take 5′ to stabilize to 37°C.
3. Weigh the animal and administer Ketamine/Xylazine mixture intraperitoneally (i.p) to anesthetize the mouse. Administer Buprenorphine i.p for pain relief. Position the mouse centrally on the heating pad. Record reflexes every 5′ to determine depth of anesthesia. Proceed to the next step only after the mouse shows a sustained loss of reflexes for 5′ accompanied by regular and stable heart rate. A pulse oximeter can be used to monitor pulse rate and blood oxygenation levels. Once mouse is anesthetized, insert the rectal probe and securely tape it to the pad.
  **IMPORTANT!** An improperly inserted or dislodged probe will result in overheating of the mouse.
  **IMPORTANT!** Anesthetics can drop body temperature. During surgery and throughout the imaging procedure, care must be taken to ensure that the core body temperature of the mouse is regulated at 37°C through the heating pad.
  **IMPORTANT!** Inducing a deep state of anesthesia in the mouse is crucial for acquiring quality images. Monitor the mouse every 5 min until it reaches a deep state of anesthesia.

### Head restraint • 10 min

4. Create a stack of blotting paper that just reaches the lower flat edge of the mounted ear bars. Add 3 drops of saline to the center of the stack to create a slight depression. The saline will keep the surgical site moist during surgery.
5. Raise the head of the mouse and slide the paper stack under the jaw. The head must rest horizontally on the slight depression in the stack. Tap the head gently with the forefinger. The head must feel as if resting firmly on the stack.
  **IMPORTANT!** If the head rests at an angle, it will be difficult to engage the ear bars. Resting the head in a horizontal position makes a larger surface area of the cranial window available for imaging.
6. Get the points of the horizontally mounted ear bars to the same level as that of the ear canal (or external auditory meatus). To achieve this, add or remove pieces of blotting paper to adjust the height of the head a few mm at a time. The points must barely touch the ears at this stage.
7. To engage an ear bar, partially unwind one of the screws and slide the ear bar gently into the ear canal a few mm at a time until the point resists. Do not force or push the ear bar. Tighten the screw. Once configured into this position, this ear bar need not be adjusted for any future imaging sessions if using the same age of mice. Follow the same process for the other ear bar. When done correctly, the head must be rested horizontally on the paper stack and feel firmly immobilized between the two ear bars.
  **IMPORTANT!** An increased heart rate (>300 beats per minute or >5 Hz if using an oximeter) during ear bar fixation is indicative of pain. Release ear bars, provide more time for anesthetic action and if that fails, administer a booster dose of anesthetic.
  **IMPORTANT!** An inadequately restrained head will cause “bobbing” of the image due to “respiration-induced movements.”

### Cranial window preparation • 25 min

8. Use cotton balls soaked in saline to liberally wet hair and skin. Make an incision in the midline of the scalp between the eyes past the ears. The scalp can be retracted using forceps and taped down to the pad or can be removed altogether as this is a non-survival procedure.
9. Dip cotton applicators in diluted 3% H_2_O_2_ and apply over the exposed skull to dislodge connective tissue. Use a scalpel to gently scrape out the periosteum and adjoining connective tissue. Sutures will appear as faint, red lines due to softening of cartilage and must be used as a guide for making incisions. Wipe away all residues of H_2_O_2_ using cotton applicators dipped in saline.
  **WARNING!** Connective tissue can cause the skin to entangle with the burr during the drilling process. This has the potential to dislodge the mouse from the restraint and cause injury to the mouse and/or the drill operator.
10. Make a circular incision of 6 mm diameter using a pneumatic dental drill between the lambdoidal suture, the sagittal suture, and the coronal suture. Hold the drill at an angle of 25° while chipping the bone. Use a round-headed 0.55″ diameter carbide drill bit [WPI, Cat. No. 501856 (#4)] to define a circular window. Begin by chipping the bone at a speed of 2500 rpm, just touching drill on the bone. Then move to a smaller, round-headed 0.47″ diameter carbide drill bit [WPI, Cat. No. 501855 (#3)] to make the incision. Gradually increase speed to a maximum of 4000 rpm to chip the bone gently over and over again until the bone just gives way or a translucent layer of dura is visible. Remove bone dust by wiping several times with kimwipes soaked in saline. Check the mouse for adequate depth of anesthesia. Administer booster doses if required.
  **WARNING!** Safety glasses must be worn by the drill operator at all times to avoid eye injury.
  **WARNING!** A P2 grade mask must be worn by the drill operator at all times to avoid inhalation of fine bone dust.
  **IMPORTANT!** Take care not to push the bone down toward the brain as minor injuries to the dura or disruption of vasculature in the cortex can provoke inflammation leading to accumulation of leukocytes. If this happens, procedural steps must be reviewed. This step requires significant surgical practice.
  **IMPORTANT!** Drilling generates fine bone dust that can deposit on the window and cloud the image. Remove dust by wiping several times with kimwipes soaked in saline.
11. For longer imaging sessions of >1.5 h duration go to step 12. Otherwise continue with step 11.
  Place a drop of saline on the window. The saline helps in separating the dura from the cranial bone while lifting the flap. Introduce the tip of a sharp, curved forceps horizontally at an angle of 10° to the edge of the bone flap. Grip the edge of the flap with the forceps. Do not push or insert the tip of the forceps into the window. Gently tug the flap and lift upwards taking care to keep the flap horizontal at all times. Next, go to step 19.

### Superfusion chamber installation • 35 min

12. Soften bone wax by warming between hands. Roll into a thin sausage. Under high magnification of a dissection microscope, place cap around circular incision and encircle the thin sausage completely around the base of the cap (Figure 3). Use the opposite end of forceps to gently mold the bone wax so that it seals the joint between the cap and the skull. The circular incision may be covered in wax at this stage. Use forceps to clear bone wax from the circular incision so that it just surrounds its outside edge.
  **IMPORTANT!** The circular incision must be kept clear from wax at all times.
13. Snap a cotton-tipped applicator into half so that its ends are pointy. Dip pointy ends into glue and allow glue to drop off the pointy end onto the outside of the cap, one drop at a time so that it completely encircles the outside of the cap and fortifies the joint between the wax and skull.
  **IMPORTANT!** Avoid putting glue on or inside the cap. The stick must pick just enough glue so they drop off the pointy end slowly one drop at a time. Too much glue can flood the cap.
14. Use another pointy swab stick to similarly apply accelerant around the outside of the cap. Accelerant reduces time for glue action. Wait 2–3 min to dry.
  **IMPORTANT!** Ensure cap is attached to the skull surface by gently pressing one of its sides down with forceps. The cap should feel firm and not get dislodged.
15. Attach the inlet and outlet polyethylene tubing to the cap. Connect the tubing to the pump-operated superfusion chamber. Adjust the operational speed of the peristaltic pump to maintain aCSF infusion at 0.3 ml/min. The cap-like reservoir should bathe in circulating aCSF.
  **IMPORTANT!** Make sure tubing is clear from wax.
16. Position the outlet tubing at an elevation of 10 cm above the mouse brain to maintain intracranial pressure at 5–8 mm Hg.
17. Under high magnification of a dissection microscope, gently puncture the thin edge of the circular incision with curved forceps, insert and lift to retract the bone flap. Clear away dura using a 30G needle. Select a section that is vascular so that dura is visible. Bleeding may occur but must cease in 2–3 min.
  **IMPORTANT!** Do not use Epinephrine or any other chemical agents to stop bleeding as chemical irritants can induce inflammation. If heavy bleeding occurs then continuing with the experiment must be re-evaluated.
18. Seal the superfusion chamber with a cover glass held in place with vacuum grease. Transfer the mouse to the microscope stage while ensuring tubing is not displaced. Now go to step 21.

### Post-surgical recovery • 5 min

19. Place 1–2 gelfoam pieces pre-moistened in saline upon the exposed brain. Remove bits that are soaked with blood, replenish with new bits. Bleeding must cease in 2–3 min.
20. Attach an 18G needle to a 5 ml syringe filled with vacuum grease. Lay a very thin circular ring of vacuum grease about 5 mm from the outer edge of the window. Remove all gelfoam bits. Place 25 μl of warm aCSF on to the exposed surface of the brain. Seal the window with a cover glass. Transfer the mouse to the microscope stage.

### Preimaging preparation • 5 min

21. To label blood plasma or study vessel permeability, administer 100 ug of dextran rhodamine (w/v) in saline intravenously.
  **IMPORTANT!** Dextran must be administered only after all bleeding has ceased. Dextran leaking out from blood vessels can coat the cerebral cortex and obstruct visualization of cortical structures.
22. Check the mouse for adequate depth of anesthesia. Administer booster doses if required.
23. Cover the body of the mouse with several pieces of heavy crepe bandage. The bandage acts as a cotton blanket. Check the core body temperature of the mouse. Position the mouse under the nose of the objective. Place a drop of water on the cover glass if using a dipping objective.

### Data acquisition • 1.5–4 H

24. Perform mouse brain imaging according to manufacturer's instructions. Mice must be monitored every 15 min for adequate depth of anesthesia. Administer booster doses as and when required. Using a fluorescence microscope, choose a region of interest (ROI). We typically use 10–20 mW of laser power for the sample with full scan field dimension (500 × 500 μm). Minor drifts during acquisition can be corrected using softwares such as Volocity.
  **IMPORTANT!** Time required for warming lasers must be taken into account before beginning an experiment. Ensure the laser is ready for use when the mouse is transferred to the stage.

### Termination of experiment • 5 min

25. As the experiments are performed on anesthetized non-recovery animals, euthanize the mouse as per institutional and/or regional animal ethics committee guidelines.

#### Procedural overview

- Presurgical preparation: 25 min
- Head restraint: 10 min
- Cranial window preparation: 25 min
- Superfusion chamber installation: 35 min
- Post-surgical recovery: 5 min
- Preimaging preparation: 5 min
- Data acquisition: 1.5–4 h
- Termination of experiment: 5 min

## Results

We applied the 2P-IBI protocol to dissect the spatio-temporal patterns of locomotion and behavior of GFP expressing leukocytes in wildtype Macgreen mice (Sasmono et al., 2003). x-y-t data were collected every 1 s and sometimes combined with 3-dimensional z stacks to create x-y-z-t time lapse images. Post-acquisition image analysis was carried out using the Volocity (Perkin Elmer) software. During analysis, firstly a unique identity code was allocated for each blood vessel within a ROI. The area of the blood vessel was derived by measuring length and breadth. The direction of blood flow within the tributaries of the blood vessel was carefully assessed. Diverging vessels with outflow of blood were classified as arteries and converging vessels with inflow of blood were classified as veins. Based on its breadth, the blood vessel was classified as a microcapillary (6–8 μm), a small (8–20 μm), or large blood vessel (>20 μm). Leukocytes were tracked as they entered the ROI and over its entire observation period. Rolling cells were defined as GFP^+^ single, round-shaped cells moving in the direction of the blood flow at a lower speed than free flowing cells. Adherent cells were defined as GFP^+^ single cells that remained stationary for 30 s or longer. The recording shows blood flowing within a pial artery (**Supplementary Movie S1**). Consistent with previous studies, leukocytes exhibited very little rolling or adherence to vessel wall (Carvalho-Tavares et al., 2000). Leukocytes appeared as “streaks” within the artery and escaped tracking due to their rapid speed. A single GFP^+^ adherent leukocyte seen here is a rare event. GFP^+^ macrophages associated with the microvasculature and meninges lining the blood vessels were also observed, as reported previously (Sasmono et al., 2003).

### Conflict of interest statement

The authors declare that the research was conducted in the absence of any commercial or financial relationships that could be construed as a potential conflict of interest.

## Supplementary material

**Movie S1 | Two-photon imaging of leukocytes within the pial vasculature of a living mouse brain.** x-y-t time-lapse sequence of a 500 × 500 μm scanfield depicts migratory patterns of leukocytes inside dextran rhodamine-labeled blood vessels. Flowing GFP^+^ cells appear as “streaks” due to their rapid speed in the blood stream. GFP (green, leukocytes), SHG (Blue, collagen), and dextran rhodamine (Red, blood vessels) were excited at 950 nm using a tunable MaiTai HP laser (Spectra Physics). Scale bar: 55 μm. All experiments dealing with live animals were performed in accordance with the relevant animal use and care guidelines and regulations.
